# Searching for the Definition of Macrosomia through an Outcome-Based Approach

**DOI:** 10.1371/journal.pone.0100192

**Published:** 2014-06-18

**Authors:** Jiangfeng Ye, Lin Zhang, Yan Chen, Fang Fang, ZhongCheng Luo, Jun Zhang

**Affiliations:** 1 MOE-Shanghai Key Laboratory of Children’s Environmental Health, Xinhua Hospital, Shanghai Jiao Tong University School of Medicine, Shanghai, China; 2 Department of Obstetrics, Xinhua Hospital, Shanghai Jiao Tong University School of Medicine, Shanghai, China; University of Cincinnati, United States of America

## Abstract

**Background:**

Macrosomia has been defined in various ways by obstetricians and researchers. The purpose of the present study was to search for a definition of macrosomia through an outcome-based approach.

**Methods:**

In a study of 30,831,694 singleton term live births and 38,053 stillbirths in the U.S. Linked Birth-Infant Death Cohort datasets (1995–2004), we compared the occurrence of stillbirth, neonatal death, and 5-min Apgar score less than four in subgroups of birthweight (4000–4099 g, 4100–4199 g, 4200–4299 g, 4300–4399 g, 4400–4499 g, 4500–4999 g vs. reference group 3500–4000 g) and birthweight percentile for gestational age (90^th^–94^th^ percentile, 95^th^-96^th^, and ≥97^th^ percentile, vs. reference group 75^th^–90^th^ percentile).

**Results:**

There was no significant increase in adverse perinatal outcomes until birthweight exceeded the 97^th^ percentile. Weight-specific odds ratios (ORs) elevated substantially to 2 when birthweight exceeded 4500 g in Whites. In Blacks and Hispanics, the aORs exceeded 2 for 5-min Apgar less than four when birthweight exceeded 4300 g. For vaginal deliveries, the aORs of perinatal morbidity and mortality were larger for most of the subgroups, but the patterns remained the same.

**Conclusions:**

A birthweight greater than 4500 g in Whites, or 4300 g in Blacks and Hispanics regardless of gestational age is the optimal threshold to define macrosomia. A birthweight greater than the 97^th^ percentile for a given gestational age, irrespective of race is also reasonable to define macrosomia. The former may be more clinically useful and simpler to apply.

## Introduction

The term “macrosomia” is used to describe a very large fetus or neonate. But there is no precise definition of macrosomia on which all obstetricians and researchers agree. Common definitions use either birthweight percentiles (e.g. P_90_ or P_97_) or birthweight cut-points (e.g. 4000g, 4500 g).[1]–[5] None however, were established based on clear clinical evidence inclusive of a broad range of perinatal outcomes.

Infant mortality decreases with increasing birthweight until birthweight reaches a certain point after which the mortality rate increases. This reverse J-shaped mortality curve has been well-described for all races. It is well-known that the weight corresponding to the lowest mortality is several hundred grams heavier than the mean birthweight.[5]–[10] Bigger babies may have survival advantages over smaller ones. But too big babies have higher morbidities of asphyxia, birth trauma, neonatal seizures, and meconium aspiration syndrome. [2], [4], [5] Macrosomic babies may also have a higher risk of adult diseases such as obesity, type 2 diabetes and cardiovascular diseases. [11]–[13] So, how big is too big? A consensus has not yet been reached to define macrosomia. [14] Given that pregnant women are now older and heavier than before, this may contribute to bigger babies. [15], [16] An evidence-based definition of macrosomia is needed.

In this study, we examined the frequently-used definitions of macrosomia through an outcome-based approach using an index of a broad range of hard-fact adverse perinatal outcomes. We aimed to answer the following questions: (1) What is an appropriate definition of macrosomia? Should it be defined based on a birthweight percentile for a given gestational age (i.e., large-for-gestational-age) or empirical birthweight cutpoints, irrespective of gestational age? (2) Does one definition fit all races?

## Materials and Methods

### Study Design, Data Source and Population Studied

We carried out a retrospective cohort study. Infants were categorized into subgroups by every 100 grams of birthweight over 4000 g or by birthweight percentile for a given gestational age at the cutoff points of the 10^th^, 25^th^, 50^th^, 75^th^, 90^th^, 95^th^, 97^th^ percentile. Perinatal mortality and morbidity were compared between subgroups with the reference groups (3500–4000 g in birthweight, or 75^th^–90^th^ percentile). The cutoff points exceeded which infants had significantly increased risks of mortality and morbidity were considered as the threshold to defined macrosomia.

This study was based on the U.S. National Center for Health Statistics (NCHS) Linked Birth-Infant Death Cohort datasets from 1995–2004. The datasets were compiled of birth and infant death certificates registered in all states, the District of Columbia, Puerto Rico, the Virgin Islands, and Guam. Each state provided to the NCHS matching birth and death certificate numbers for each infant less than one year of age occurring in a given calendar year. NCHS used the matching certificate numbers to extract record from the NCHS statistical files and link these data to establish the linked record file. The methodological details of the linkage of birth and death record were published in the Technical Notes of National Vital Statistical Reports. [17] The data are coded according to uniform specification and comply with uniform quality control standard. The data are publicly accessible at the Centers for Disease Control and Prevention Web site. Available information in these files included demographic characteristics of mothers, obstetric history, current pregnancy, labor and delivery complications and birth outcomes.

For the purpose of this analysis, the study sample was restricted to: 1) race of non-Hispanic White, non-Hispanic Black, and Hispanic; 2) singleton pregnancies; 3) birthweight ≥500 grams; and 4) term births (gestational age 37–42weeks). Infants had missing birthweight or gestational age were excluded. Birthweight >5500 grams was considered implausible and treated as missing value. [18] Asian and other races were excluded due to the small number of deaths.

### Definition of Covariates

Race and ethnic origin were based on self-report. Maternal age at delivery was grouped into: <19, 20–34 and ≥35 years old. Mother’s marital status was classified as: married, unmarried and unknown. Education levels were recoded as: <12 years (less than high school), 12 years (high school), 13–16 years (college), ≥17 years (graduate school). Smoking during pregnancy was defined by average number of cigarettes per day. We recoded this variable as “nonsmokers” (0 cigarettes per day), “light smokers” (1 to 10 cigarettes per day) and “heavy smokers” (more than 10 cigarettes per day). Information describing “when the prenatal care started” was classified as: 1^st^ trimester (1^st^-3^rd^ month), 2^nd^ trimester (4^th^-6^th^ month), 3^rd^ trimester (7^th^-9^th^ month) and no prenatal care. Mode of delivery was classified as vaginal or cesarean delivery.

### Estimation of Gestational Age

Gestational age was recorded firstly based on self-reported last menstrual period (LMP) and secondly by clinical estimate (CE). Limitations of LMP-based estimate have been well documented. [19], [20] Several methods have been proposed to reduce misclassifications of LMP-based gestational age. Qin and colleagues [21] recently proposed that CE of gestational age substitutes for LMP-based gestational age when the difference between the two estimates was greater than two weeks. Compared to other techniques, this method almost eliminated the aberrant second mode of gestational age distribution, and was demonstrated to be effective in correcting large errors in gestational age estimates. A further benefit is that records are reclassified rather than excluded altogether. Thus, we adopted LMP/CE method to estimate gestational age.

### Study Outcomes

The main outcomes included stillbirth, neonatal death and 5-min Apgar score less than four. Stillbirth included all fetal deaths with a birthweight of 500 g or more. Neonatal death included infant deaths from 0 to 27 days after birth. As fetal and neonatal mortality are rare outcomes, a composite perinatal mortality and morbidity index (PMMI) including stillbirth, neonatal death and a 5-min Apgar score less than four was created. In the exploratory analyses we observed that infants did not have significantly increased risk of postnatal death even with a birthweight of 4500 or more. Therefore, postnatal mortality was not considered as one of the outcome measures.

### Ethics Statement

Data for this analysis were obtained from anonymized data rendering ethical approval unnecessary by the Shanghai Xinhua Hospital Research Ethics Board.

### Statistical Analyses

Two types of definition of macrosomia were compared - based on empirical birthweight or statistical distribution of birthweight. The definitions were examined through an outcome-based approach with the assumption that birthweight-specific infant mortality curve follows a reversed J-shape. [6]–[10] Macrosomia is defined as weights or weight percentiles that exceed the nadir of the mortality curve. Infants were categorized into subgroups by birthweight percentile cutoffs: 75^th^, 90^th^, 95^th^ and 97^th^ for each gestational week. Birthweight percentiles by gestational age were calculated according to the global reference for fetal-weight and birthweight percentiles by race/ethnicity. [22] Table 1 contains birthweight percentiles by gestational age for non-Hispanic White, non-Hispanic Black and Hispanics. The risks of perinatal mortality and morbidity were compared between subgroups. Exploratory analyses observed that birthweight corresponding to the lowest morbidity and mortality was several hundred grams heavier than mean birthweight, as in previous studies.[6]–[10] Subgroups with birthweight at 75^th^–90^th^ percentiles or 3500–4000 g corresponded to the nadir of birthweight-specific mortality curve and, therefore, were used as the reference categories. Given that this study focuses on macrosomia, we presented the main results for birthweight greater than 3500 g or 75th percentile in separate analyses.

**Table 1:** Race-specific birthweight distributions in singleton term births, U.S. 1995–2004.

Gestational age (weeks)	N^a^	Birthweight (g)				Birthweight percentile			
		Mean (SD)	4000–4499 (%)	4500–4999(%)	≥5000(%)	75^th^–89^th^ (%)	90^th^–94^th^ (%)	95^th^–96^th^ (%)	≥97^th^ (%)
**White**	19748437	3465(476)	10.6	1.7	0.2	15.4	5.5	2.5	6.0
37	1608614	3154(484)	3.7	0.6	0.1	16.9	7.5	3.6	12.4
38	3622784	3336(461)	6.3	0.9	0.1	16.4	7.2	3.3	9.6
39	5299598	3462(448)	9.4	1.4	0.1	16.1	5.7	2.5	6.3
40	6020734	3563(448)	13.2	2.1	0.2	15.0	4.5	2.2	3.7
41	2398201	3631(468)	16.7	3.2	0.3	12.1	3.8	1.3	2.7
42	798506	3571(490)	14.6	3.0	0.4	15.3	5.2	2.0	3.0
**Black**	4525824	3259(474)	5.1	0.8	0.1	14.8	5.7	2.6	7.2
37	486874	3004(481)	2.1	0.4	0.1	15.7	8.1	4.0	14.0
38	918529	3152(457)	3.1	0.5	0.1	16.8	7.0	3.2	10.1
39	1182564	3267(448)	4.5	0.7	0.1	14.8	5.9	2.6	7.0
40	1280923	3365(450)	6.6	0.9	0.1	13.9	4.7	1.9	4.9
41	474383	3420(473)	8.9	1.3	0.2	12.2	3.6	1.8	3.2
42	182551	3346(487)	7.2	1.1	0.1	15.3	5.0	2.0	3.0
**Hispanic**	6563032	3397(464)	8.0	1.2	0.2	14.9	6.2	2.5	6.9
37	577094	3152(484)	3.6	0.6	0.1	16.5	8.8	4.2	15.9
38	1242509	3290(453)	5.1	0.8	0.1	17.3	7.6	3.6	10.7
39	1779751	3398(441)	7.2	1.0	0.1	15.8	6.8	2.6	6.7
40	1905498	3483(443)	9.8	1.5	0.2	13.9	5.1	1.9	4.3
41	772771	3539(460)	12.5	2.1	0.2	10.9	3.8	1.3	2.9
42	285409	3489(474)	34.5	11.0	2.0	14.5	5.4	2.2	3.0

a Number of live births.

Multivariable logistic regression was used to estimate odds ratios (ORs) of perinatal mortality and morbidity. We used OR = 2 as the pre-defined criterion to identify clinically important macrosomia similar to the definition of fetal growth restriction by neonatal death risk in Boulet’s study. [23] The analysis of risk of infant mortality and morbidity was adjusted for maternal age, parity, infant sex and gestational age, maternal diabetes, chronic hypertension, pregnancy associated hypertension, eclampsia, smoking and social economic status (marital status, education) and prenatal care. The selection of the covariates included in the models was based on findings in the literature. All variables were retained in the model regardless of statistical significance as they had a prior theoretical association with the outcomes. All analyses were carried out using SAS version 9.2 (SAS Institute Inc, Cary, NC).

## Results

There were 39,956,864 live births and 539,915 stillbirths in the linked dataset. A total of 30,831,694 live births and 38,053 stillbirths were included. There were large variations in the birthweight distribution among infants of different races (Table 1). About 12% of Whites, 6% of Blacks and 9% of Hispanics had a birthweight greater than 4000 g. A total of 1.9% of Whites, 0.9% of Blacks and 1.4% of Hispanics weighed heavier than 4500 g at birth. The prevalence of birthweight greater than the 97^th^ percentile was 6.0%, 7.2% and 6.9% for White, Black and Hispanic, respectively.

Table 2 shows the prevalence of adverse perinatal outcomes. Cesarean delivery rates were around 20% in all the three races, and increased with higher birthweight. Prevalence of stillbirth, neonatal death and 5-min Apgar score less than four decreased with higher birthweight. Infants with birthweight of 3500–4000 g had the lowest perinatal mortality and morbidity. When the birthweight increased further, prevalence of perinatal mortality and morbidity increased. Table 3 shows that the risk of perinatal mortality increased slightly when birthweight exceeded the 95^th^ in all the three races. When birthweight exceeded the 97^th^ percentile, the risk increased further. The adjusted odds ratios (aORs) of perinatal mortality were 1.39 (95%CI:1.28,1.50), 1.64 (95%CI:1.46,1.85) and 1.58 (95%CI: 1.39,1.80) in Whites, Blacks and Hispanics, respectively. A similar trend was found in the risk of 5-min Apagr score less than four and composite index of morbidity and mortality.

**Table 2:** Prevalence of perinatal and neonatal adverse outcomes in singleton birth cohort, U.S. 1995–2004.

Gestational age (weeks)	Cesareandelivery (%)	Stillbirth^b^(per 1000)	Neonatal death(per 1000)	5-min Apgar score less than four(per 1000)
**White (N** a ** = **19748437)	21.5	1.1	1.0	1.0
<2500	29.1	11.7	11.1	3.5
2500–2999	20.6	2.2	1.8	1.3
3000–3499	19.5	0.9	0.8	0.9
3500–3999	21.3	0.5	0.5	0.9
4000–4499	26.8	0.6	0.5	1.1
4500–4999	36.7	1.2	0.7	1.6
≥5000	50.2	4.7	1.9	4.4
**Black (N** a ** = **4525824)	22.9	1.7	1.3	1.8
<2500	24.9	9.9	7.2	4.7
2500–2999	19.6	1.9	1.6	1.9
3000–3499	21.0	1.0	0.9	1.4
3500–3999	25.9	1.0	0.8	1.5
4000–4499	35.3	1.8	0.9	2.3
4500–4999	48.6	5.4	1.5	4.5
≥5000	62.2	18.5	5.0	12.9
**Hispanic (N** a ** = **6563032)	21.5	1.3	1.0	1.0
<2500	26.7	13.7	10.8	2.8
2500–2999	18.8	2.0	1.5	1.1
3000–3499	19.1	0.9	0.7	0.9
3500–3999	22.6	0.7	0.5	0.9
4000–4499	30.4	1.0	0.5	1.2
4500–4999	42.9	2.8	0.9	2.7
≥5000	58.3	9.9	2.5	6.3

a Number of live births.

b Based on live births plus stillbirths.

**Table 3:** Risks of perinatal mortality and morbidity (the occurrence of stillbirth, neonatal death, or 5-min Apgar score <4) by birthweight percentile (excluding deaths due to congenital anomalies).

Birthweightpercentile	Mortality^a^			5-min Apgar score less than four			Perinatal mortality and morbidity^c^		
	Prevalence(per 1000)	adjusted OR(95% CI)^b^	p value	Prevalence(per 1000)	adjusted OR(95% CI)^b^	p value	Prevalence(per 1000)	adjusted OR(95% CI)^b^	p value
**White**		N = 16048878							
P_75_–P_89_	0.9	1.00		0.9	1.00		1.7	1.00	
P_90_–P_94_	0.9	1.03(0.94,1.13)	0.5134	1.0	1.06(0.98,1.15)	0.1532	1.8	1.04(0.97,1.11)	0.2430
P_95_–P_96_	1.0	1.08(0.95,1.22)	0.2403	1.1	1.24(1.12,1.38)	<0.0001	1.9	1.15(1.06,1.26)	0.0009
**≥P_97_**	**1.4**	**1.39(1.28,1.50)**	**<0.0001**	1.3	1.40(1.30,1.50)	<0.0001	**2.5**	**1.36(1.28,1.43)**	**<0.0001**
**Black**		N = 3758701							
P_75_–P_89_	1.3	1.00		1.5	1.00		2.7	1.00	
P_90_–P_94_	1.5	1.03(0.88,1.2)	0.7312	1.5	1.01(0.88,1.15)	0.9323	2.9	1.00(0.90,1.11)	0.9610
P_95_–P_96_	1.9	1.24(1.03,1.51)	0.0263	1.7	1.14(0.95,1.35)	0.1503	3.3	1.13(0.98,1.29)	0.0853
**≥P_97_**	**3.0**	**1.64(1.46,1.85)**	**<0.0001**	2.5	1.64(1.47,1.82)	<0.0001	**5.2**	**1.59(1.47,1.73)**	**<0.0001**
**Hispanic**		N = 6564283							
P_75_–P_89_	1.0	1.00		0.9	1.00		1.4	1.00	
P_90_–P_94_	1.0	0.96(0.81,1.13)	0.5827	0.9	1.04(0.86,1.27)	0.6562	1.4	1.03(0.91,1.16)	0.6888
P_95_–P_96_	1.1	0.92(0.73,1.16)	0.4584	0.9	0.98(0.74,1.31)	0.9132	1.4	0.90(0.75,1.08)	0.2483
**≥P_97_**	**2.1**	**1.58(1.39,1.80)**	**<0.0001**	1.6	1.74(1.48,2.05)	<0.0001	**2.8**	**1.65(1.49,1.82)**	**<0.0001**

a The occurrence of stillbirth or neonatal death.

b Data are adjusted ORs estimated from multiple regression models adjusted for maternal age, gestational age, parity, infant sex, maternal diabetes, chronic hypertension, pregnancy associated hypertension, eclampsia, smoking, social economic status (marital status, education) and month of prenatal care started.

c The occurrence of stillbirth, neonatal death, or 5-min Apgar score less than four.

When infants weighing between 4000–4500 g were further classified into every 100 g subgroups, we found that the aORs increased gradually with higher birthweight. When birthweight exceeded 4500 g, the risk of perinatal mortality and morbidity increased substantially in Whites (aOR 1.91 and 1.94, respectively) (Table 4). However, in Blacks, the aORs of adverse outcomes exceeded 2.0 when birthweight was heavier than 4300 g (aOR = 2.04 for 5-min Apgar score less than four). In Hispanci, the aOR exceeded 2.0 when birthweight was heavier than 4300 g (aORs 2.03 for 5-min Apgar score less than four). The aORs of composite index of mortality and morbidity exceeded 2.0 when birthweight exceeded 4500 g in Blacks and Hispanics (aOR 3.09 and 2.71, respectively). We did two sensitive analyses by restricting the analyses to vaginal deliveries (Table S1 and S2) and vaginal deliveries excluding vaginal births after cesarean section (Table S3 and S4), the aORs of perinatal morbidity and mortality were larger for most of the subgroups, but the patterns remained the same.

**Table 4:** Risks of perinatal mortality and morbidity (stillbirth, neonatal death, or 5-min Apgar score <4) by birthweight (excluding deaths due to congenital anomalies).

Birthweight	Mortality^a^				5-min Apgar score less than four				Perinatal mortality and morbidity^c^		
	Prevalence (per 1000)	adjusted OR (95% CI)^b^	p value		Prevalence (per 1000)	adjusted OR (95% CI)^b^	p value		Prevalence (per 1000)	adjusted OR (95% CI)^b^	p value
**White**		N = 16048878									
3500–3999	0.8	1.00			0.9	1.00			1.6	1.00	
4000–4099	0.8	1.08(0.96,1.21)	0.2067		1.0	1.20(1.09,1.31)	0.0001		1.7	1.12(1.04,1.21)	0.0025
4100–4199	0.9	1.20(1.07,1.34)	0.0021		1.1	1.21(1.10,1.33)	<0.0001		1.8	1.22(1.13,1.31)	<0.0001
4200–4299	1.0	1.34(1.17,1.54)	<0.0001		1.0	1.14(1.01,1.29)	0.0393		1.8	1.21(1.1,1.33)	0.0001
4300–4399	1.0	1.43(1.24,1.65)	<0.0001		1.2	1.33(1.18,1.51)	<0.0001		2.1	1.37(1.24,1.5)	<0.0001
4400–4499	1.0	1.47(1.22,1.77)	<0.0001		1.2	1.36(1.16,1.60)	0.0001		2.1	1.38(1.21,1.57)	<0.0001
**4500–4999**	1.5	**1.91(1.69,2.15)**	<0.0001		1.6	1.77(1.60,1.96)	<0.0001		2.9	**1.82(1.68,1.98)**	<0.0001
≥5000	6.0	7.23(5.98,8.76)	<0.0001		4.4	4.73(3.89,5.75)	<0.0001		9.5	5.61(4.86,6.48)	<0.0001
**Black**		N = 3758701									
3500–3999	1.4	1.00			1.5	1.00			2.8	1.00	
4000–4099	1.6	1.10(0.86,1.41)	0.4391		1.8	1.16(0.94,1.41)	0.1612		3.2	1.10(0.94,1.29)	0.2454
4100–4199	2.0	1.27(1.00,1.62)	0.0484		2.3	1.55(1.29,1.87)	<0.0001		4.0	1.44(1.24,1.68)	<0.0001
4200–4299	2.0	1.16(0.84,1.60)	0.3715		2.7	1.80(1.44,2.25)	<0.0001		4.4	1.51(1.25,1.83)	<0.0001
**4300–4399**	2.6	1.67(1.24,2.23)	0.0006		3.0	1.94(1.54,2.45)	<0.0001		5.3	**1.83(1.52,2.21)**	<0.0001
4400–4499	3.9	**2.35(1.69,3.27)**	<0.0001		2.3	1.46(1.02,2.08)	0.0394		5.8	1.82(1.42,2.34)	<0.0001
4500–4999	5.8	3.49(2.86,4.27)	<0.0001		4.5	2.89(2.39,3.49)	<0.0001		9.5	3.09(2.68,3.57)	<0.0001
≥5000	18.7	9.68(7.09,13.22)	<0.0001		12.9	7.63(5.52,10.53)	<0.0001		35.9	8.33(6.59,10.54)	<0.0001
**Hispanic**		N = 6564283									
3500–3999	0.9	1.00			0.9	1.00			1.3	1.00	
4000–4099	1.1	1.27(1.03,1.56)	0.0258		0.9	1.07(0.83,1.39)	0.6056		1.4	1.19(1.01,1.4)	0.0403
4100–4199	1.1	1.10(0.87,1.39)	0.4158		1.3	1.57(1.25,1.98)	0.0001		1.7	1.36(1.15,1.6)	0.0002
4200–4299	1.8	1.94(1.54,2.45)	<0.0001		1.3	1.51(1.12,2.04)	0.0069		2.2	1.64(1.36,1.99)	<0.0001
**4300–4399**	1.7	1.68(1.29,2.18)	0.0001		1.6	1.81(1.35,2.43)	<0.0001		2.4	1.79(1.48,2.17)	<0.0001
4400–4499	1.6	1.44(1.00,2.09)	0.0519		1.1	1.25(0.78,2.00)	0.3493		2.1	1.42(1.06,1.88)	0.0173
**4500–4999**	3.4	**2.69(2.19,3.29)**	<0.0001		2.7	2.96(2.35,3.71)	<0.0001		4.4	**2.71(2.33,3.17)**	<0.0001
≥5000	11.4	9.14(6.7,12.46)	<0.0001		6.3	7.09(4.62,10.88)	<0.0001		13.7	7.89(6.11,10.2)	<0.0001

a The occurrence of stillbirth or neonatal death.

b Data are adjusted ORs estimated from multiple regression models adjusted for maternal age, gestational age, parity, infant sex, maternal diabetes, chronic hypertension, pregnancy associated hypertension, eclampsia, smoking, social economic status (marital status, education) and month of prenatal care started.

c The occurrence of stillbirth, neonatal death, or 5-min Apgar score less than four.

## Discussion

Our findings suggest that the term macrosomia should be defined as a birthweight greater than 4500 g regardless of gestational age in Whites, or as greater than the 97^th^ percentile in birthweight for gestational age for three races. In general, our study supported the AJOG’s definition of macrosomia as 4500 g or more for Whites. [3] However, in Blacks and Hispanics, the optimal threshold to define macrosomia may be 200 g lower, at 4300 g based on the perinatal mortality and 5-min Apgar score. The definition based on birthweight may be more clinically useful and simpler to apply than that based on birthweight percentile.

We observed no significant increase in adverse perinatal outcomes in the subgroup of 90^th^-94^th^ and 95^th^-96^th^ percentiles in birthweight. Our finding doesn’t support the definition of macrosomia as LGA. The 97^th^ percentile for a given gestational week may be a better cutoff point to define what is too big in birth size. Based on the pre-defined criterion (OR = 2 as the cutoff point) to identify clinically important macrosomia [23], even the 97^th^ percentile could not meet this criterion.

Several studies using perinatal mortality and morbidity as outcomes to examine the impact of macrosomia reported somewhat similar conclusions. Boulet SL et al [2] used the NCHS database from 1995–1997 and found that although a definition of macrosomia as >4000 g may be useful for the identification of increased risk of labor, >4500 g may be more predictive of neonatal morbidity, and >5000 g may be a better indicator of infant mortality risk. Although there is no general consensus on the choice of optimal outcome indicators in defining clinically significant macrosomia, stillbirth and neonatal death were the most frequently used outcomes. [24]–[26] The 5-min Apgar score less than four is strongly predictive of neonatal death. The mortality of neonatal death in term infants with five-minute Apgar score less than four was more than one in five (244 per 1000) infants.

We found that risks of perinatal and infant mortality and morbidity increased gradually in the infants weighted between 4000–4500 g in Whites. Once birthweight exceeded 4500 g, risks elevated substantially. Our findings further are in general agreement with previous study and support the American Congress of Obstetricians and Gynecologists’ definition of macrosomia as 4500 g or more. [3], [5] However, our study also showed that there was some variation among races/ethnicities. Birthweight at 4500 g appears to be a good cutoff point for Whites, but in Blacks and Hispanics, the cutoff seems about 200 g lower.

Our study also found that the aORs for babies with birthweight greater than 4500 g were substantially larger than those above the 97^th^ percentile. This may be explained by the fact that before 40 weeks, 97^th^ percentiles of birthweight correspond to birthweight much lower than 4500 g. Thus, the >97^th^ percentile group included a substantial proportion of births that are reasonable in size. Given this deficiency, a definition of macrosomia based on 4500 g for Whites, and 4300 g for Blacks and Hispanics is recommended.

In Pasupathy’s study [27], neonatal outcomes of macrosomia defined by customized centiles, population centiles and birthweight greater than 4000 g were compared in Whites. They suggested customized standard as the better definition of LGA than population centiles or a birthweight of 4000 g for its stronger association with adverse neonatal outcomes. The macrosomia infants defined by the combination of customized centile/population centile or customized centile/empirical birhtweight of 4000 g in Pasupathy’s study had birthweight ranging from 4020 g to 4475 g and from 4160 g to 4520 g, respectively, which were a little lower than that defined by the birthweight of 4500 g or more. The subgroup of infants defined as macrosomia in the current study (birthweight 4500 or more) had twofold increased risk of perinatal mortality and morbidity compared with the reference group (3500–3999 g), which was similar to that of macrosomia defined by the combined definition in Pasupathy’s study (aOR = 1.8 and 1.9, respectively). For simplicity in clinical application, we would suggest the definition of macrosomia as a birthweight of 4500 g or more, irrespective of gestational age among Whites.

Cesarean section is an effective intervention to reduce the risks of neonatal adverse outcomes when it is medically justified. The true risk of adverse perinatal outcomes in macrosomia may be underestimated when medical necessary cesarean section was available. [5], [28] In our study, rates of cesarean delivery was around 20% and increased significantly when birthweight was higher than the 90^th^ percentile or 4000 g. When we restricted the analyses to vaginal deliveries, the aORs of adverse outcomes for big babies were larger, but the cutoff point remained essentially unchanged. Even after excluding vaginal births after cesarean section, the risks of neonatal mortality and morbidity increased with higher birthweight. The increase trends of adverse perinatal outcomes in macrosomia infants may not be explained by the confounding of mode of delivery.

### Limitations

Our study has several potential limitations. First, the estimate of gestational age may not be accurate in some babies, which might have resulted in some misclassifications in birthweight percentiles, and consequently, reduced the aORs for the extreme weight (>90^th^ or 97^th^) percentile groups. Secondly, high prepregnancy body mass index (BMI) is known to be associated with both macrosomia and adverse birth outcomes [29], [30], but maternal prepregnancy BMI was not available. We speculate that adjusting for prepregnancy BMI may decrease the ORs, especially in races/ethnicities where obesity is more prevalent. However, the trend should remain the same, and thus, this deficiency should not materially affect the main findings. Thirdly, cesarean delivery rate was around 20% for all three races. Given that cesarean section is an effective intervention to reduce maternal and neonatal mortality when it is medically justified [28], [31]–[33], the risk of macrosomia without intervention may be underestimated. The NCHS files do not include information on the timing or indication of cesarean delivery. Thus, the impact of cesarean section on macrosomia is unclear. We analyzed the data by including all births and, then, vaginal deliveries only. We realize that this analytic approach may not totally address the issue of confounding by indication, and the risk of mortality in macrosomia might have been greater without cesarean delivery. However, we found that the findings from vaginal deliveries were consistent with those for all births. Fourthly, the risks of adverse outcomes fluctuated among Hispanic births with birthweight greater than 4200 g. Similar fluctuations appeared in the vaginal births even after adjustment for maternal age, gestational age, parity, infant sex, maternal diabetes, social economic status, etc. The fluctuation in the risk of adverse outcomes in Hispanic infants may be explained by the confounding of maternal obesity. But there was no information of maternal anthropometric indices in this dataset. The absence of information on maternal height and weight prevented us from controlling for the confounding of maternal obesity for the association between macrosomia and adverse perinatal outcomes. Finally, babies with macrosomia may have higher risk of adult diseases, such as obesity, diabetes and cardiovascular diseases. [11]–[13] The reverse J-shape relationship may also apply to the relationship birthweight and adult diseases. The absence of information on long-term outcomes in the NCHS files prevented us from consideration of long-term effects of large birth size.

### Implications

Birthweight at 4500 g may be the optimal cutoff point to define macrosomia in Whites, but in Blacks and Hispanics, the cutoff point seems to be 4300 g. The definition based on birthweight irrespective of gestational age may be more clinically useful than the one based on birth weight for gestational age. Application of this pragmatic definition may be helpful to improve intrapartum management.

## Supporting Information

Table S1 Risks of perinatal morbidity and mortality (the occurrence of stillbirth, neonatal death, or 5-min Apgar score <4) by birthweight percentile (excluding deaths due to congenital anomalies) in vaginal deliveries.
(DOCX)

Table S2 Risks of perinatal morbidity and mortality (stillbirth, neonatal death, or 5-min Apgar score <4) by birthweight (excluding deaths due to congenital anomalies) in vaginal deliveries.
(DOCX)

Table S3 Risks of perinatal morbidity and mortality (the occurrence of stillbirth, neonatal death, or 5-min Apgar score <4) by birthweight percentile (excluding deaths due to congenital anomalies) in vaginal deliveries without previous cesarean section.
(DOCX)

Table S4 Risks of perinatal morbidity and mortality (stillbirth, neonatal death, or 5-min Apgar score <4) by birthweight (excluding deaths due to congenital anomalies) in vaginal deliveries without previous cesarean section.
(DOCX)
