# Supplementary material for: Searching for the Definition of Macrosomia through an Outcome-Based Approach
Source: PLoS One. 2014 Jun 18;9(6):e100192. doi: 10.1371/journal.pone.0100192 (PMC4062533; doi:10.1371/journal.pone.0100192)
Supplement: Table S1 — Risks of perinatal morbidity and mortality (the occurrence of stillbirth, neonatal death, or 5-min Apgar score <4) by birthweight percentile (excluding deaths due to congenital anomalies) in vaginal deliveries. (DOCX) [file pone.0100192.s001.docx]

Table S1 Risks of perinatal morbidity and mortality (the occurrence of stillbirth, neonatal death, or 5-min Apgar score <4) by birthweight percentile (excluding deaths due to congenital anomalies) in vaginal deliveries

|  | **Neonatal mortality** | | |  | **5-min Apgar score less than four** | | |  | **Neonatal mortality and morbidity^b^** | | |
| --- | --- | --- | --- | --- | --- | --- | --- | --- | --- | --- | --- |
| **Birthweight percentile** | **Prevalence (per 1000)** | **adjusted OR (95% CI)^a^** | **p value** |  | **Prevalence (per 1000)** | **adjusted OR (95% CI)^a^** | **p value** |  | **Prevalence (per 1000)** | **adjusted OR (95% CI)^a^** | **p value** |
| **White** |  | N=12544613 |  |  |  |  |  |  |  |  |  |
| P_75_-P_89_ | 0.2 | 1.00 |  |  | 0.7 | 1.00 |  |  | 0.9 | 1.00 |  |
| P_90_-P_94_ | 0.3 | 1.09(0.91,1.30) | 0.3694 |  | 0.8 | 1.14(1.02,1.27) | 0.0176 |  | 1.0 | 1.12(1.02,1.23) | 0.0208 |
| P_95_-P_96_ | 0.2 | 1.00(0.78,1.29) | 0.9857 |  | 0.9 | 1.36(1.19,1.56) | <.0001 |  | 1.1 | 1.25(1.1,1.41) | 0.0005 |
| >=P_97_ | 0.3 | 1.13(0.95,1.35) | 0.1690 |  | 1.2 | 1.72(1.57,1.89) | <.0001 |  | 1.4 | 1.50(1.38,1.64) | <.0001 |
| **Black** |  | N=2887312 |  |  |  |  |  |  |  |  |  |
| P_75_-P_89_ | 0.4 | 1.00 |  |  | 1.2 | 1.00 |  |  | 1.6 | 1.00 |  |
| P_90_-P_94_ | 0.4 | 0.92(0.68,1.24) | 0.5653 |  | 1.2 | 0.97(0.82,1.16) | 0.7752 |  | 1.5 | 0.92(0.79,1.07) | 0.2916 |
| P_95_-P_96_ | 0.5 | 1.22(0.84,1.77) | 0.2888 |  | 1.5 | 1.21(0.97,1.52) | 0.0918 |  | 1.8 | 1.15(0.94,1.4) | 0.1738 |
| >=P_97_ | 0.5 | 1.14(0.88,1.49) | 0.3153 |  | 2.1 | 1.69(1.47,1.95) | <.0001 |  | 2.4 | 1.44(1.27,1.64) | <.0001 |
| **Hispanic** |  | N=2350735 |  |  |  |  |  |  |  |  |  |
| P_75_-P_89_ | 0.2 | 1.00 |  |  | 0.7 | 1.00 |  |  | 0.9 | 1.00 |  |
| P_90_-P_94_ | 0.2 | 1.10(0.71,1.72) | 0.6721 |  | 0.8 | 1.10(0.86,1.4) | 0.4415 |  | 1.0 | 1.13(0.91,1.40) | 0.2635 |
| P_95_-P_96_ | 0.2 | 0.98(0.50,1.92) | 0.9633 |  | 0.7 | 0.98(0.68,1.41) | 0.9081 |  | 0.8 | 0.96(0.69,1.34) | 0.8108 |
| >=P_97_ | 0.3 | 1.50(1.01,2.24) | 0.0454 |  | 1.5 | 2.02(1.65,2.48) | <.0001 |  | 1.7 | 1.89(1.57,2.27) | <.0001 |

a Data are adjusted ORs estimated from multiple regression models adjusted for maternal age, gestational age, parity, infant sex, maternal diabetes, chronic hypertension, pregnancy associated hypertension, eclampsia, smoking, social economic status (marital status, education) and month of prenatal care started.

b The occurrence of neonatal death or 5-min Apgar score less than four
